# Mutual role of ecto-5'-nucleotidase/CD73 and concentrative nucleoside transporter 3 in the intestinal uptake of dAMP

**DOI:** 10.1371/journal.pone.0223892

**Published:** 2019-10-21

**Authors:** Katsuya Narumi, Tsukika Ohata, Yuichi Horiuchi, Hiroshi Satoh, Ayako Furugen, Masaki Kobayashi, Ken Iseki

**Affiliations:** 1 Laboratory of Clinical Pharmaceutics & Therapeutics, Division of Pharmasciences, Faculty of Pharmaceutical Sciences, Hokkaido University, Sapporo, Hokkaido, Japan; 2 Research and Development Division, Hokkaido Research Institute, Nissei Bio Co. Ltd, Eniwa, Hokkaido, Japan; 3 Department of Pharmacy, Hokkaido University Hospital, Sapporo, Hokkaido, Japan; Forman Christian College (A Chartered University), Lahore, Pakistan, PAKISTAN

## Abstract

2'-Deoxyadenosine 5'-monophosphate (dAMP), a deoxyribonucleotide found in DNA, affects intestinal cell growth. The molecular mechanisms underlying gastrointestinal absorption of foreign DNA ingested along with food has hardly been investigated. The aim of this study was to investigate the mechanism underlying intestinal absorption of dAMP. The uptake of [^3^H]dAMP by Caco-2 cells was Na^+^- and pH-dependent and was inhibited by various nucleosides. In contrast, nitrobenzylthioinosine (NMBPR), an equilibrative nucleoside transporter inhibitor, showed little inhibitory effects on [^3^H]dAMP uptake. Additionally, human concentrative nucleoside transporter (CNT) 3, transiently expressed in COS-7 cells, mediated the uptake of [^3^H]dAMP. A kinetic study revealed that the *K*_m_ value of CNT3-mediated uptake of dAMP (59.6 μM) was close to that of 2'-deoxyadenosine (dAdo) (56.3 μM), whereas the dAMP *V*_max_ (15.6 pmol·mg protein^–1^min^–1^) was 500-fold lesser than the dAdo *V*_max_ (7782 pmol·mg protein^–1^min^–1^). Further, [^3^H]dAMP uptake was greater in COS-7 cells expressing ecto-5'-nucleotidase/CD73 with CNT3 than in those expressing CNT3 alone. These data suggest that, although dAMP is a substrate of CNT3, it is dephosphorylated to dAdo by CD73 and is efficiently absorbed as dAdo from the intestinal lumen.

## Introduction

Dietary nucleic acid and nucleotides play important roles in intestinal development and recovery [1]. Holen et al. have reported that 2'-deoxyadenosine 5'-monophosphate (dAMP), a deoxyribonucleotide found in DNA, affects intestinal cell growth [2]. Although the ingestion of nucleic acids as a nutritional supplement has attracted considerable attention of the researchers recently, the fate of foreign DNA ingested with daily food intake in the mammalian gastrointestinal tract is not fully understood yet.

Most dietary nucleotides are ingested mainly as nucleoproteins present in all forms of food of animal and vegetable origin. In general, nucleoproteins and nucleic acids (DNA or RNA) are degraded into nucleotides by proteases and nucleases in the gut. Subsequently, the nucleotides are dephosphorylated by phosphatases and nucleotidases in the lumen and efficiently absorbed as nucleosides and nucleobases [3,4]. Although Sánchez-Pozo and Gil have suggested that nucleotides are incorporated into tissues, it is still debatable whether nucleotides can be fully absorbed through the gut wall [5,6].

Nucleosides are relatively hydrophilic molecules and require specific transport proteins for permeation through the cell membranes. Two classes of nucleoside transporter proteins mediate nucleoside transport across the cellular membranes, namely, Na^+^-dependent concentrative nucleoside transporters (CNTs) and Na^+^-independent equilibrative nucleoside transporters (ENTs) [7,8]. The CNT family has three members (CNT1-3) with different substrate specificity. CNT1 prefers transport of the pyrimidine nucleosides, whereas CNT2 prefers transport of the purine-nucleosides. On the other hand, CNT3 shows a broader substrate selectivity, accepting both purine and pyrimidine nucleosides. Interestingly, CNT3 is a Na^+^-dependent transporter but can work as an H^+^-coupled transporter in the absence of extracellular Na^+^ [9]. In mammals, four ENTs (ENT1-4) have been characterized till date. ENT1, ENT2, and ENT4 are primarily found in the plasma membrane, whereas ENT3 is distributed mainly within the endomembrane system [10]. ENT1 and ENT2 have broad substrate specificity and tissue distribution. ENT1 is selectively inhibited by nanomolar concentrations of nitrobenzylthioinosine (NBMPR), whereas ENT2 is resistant to NBMPR at up to a concentration of 1 μM [11]. ENT4 was first characterized as a monoamine transporter, and its activity has been reported to increase highly in an acidic pH [12,13]. The polarized intestinal epithelial cells have CNTs on the apical membrane and ENTs on the basolateral membrane [14,15]. Although these transporters were shown to be involved in the transport of nucleobases, nucleosides, and nucleoside analogs, there is no report specifying whether nucleotides (i.e., nucleoside phosphates) are their substrates or not.

Ecto-5’-nucleotidase (ecto-5’-NT; CD73) is expressed abundantly on the apical surface of cultured and primary intestinal epithelial cells and functions in hydrolyzing extracellular nucleoside monophosphate into bioactive nucleoside intermediates [16,17]. CD73 has been reported to influence the control of a variety of physiologic responses, including ischemic preconditioning, tissue injury, platelet function, hypoxia, inflammation, and cancer progression [18–20]. Little is, however, known whether CD73 has a physiological role in the intestinal absorption of deoxyribonucleotide. This study characterized the mechanisms of intestinal absorption of deoxyribonucleotide, especially dAMP, and provided evidence for the mutual role of CD73 and CNT3 in the cellular uptake of dAMP and 2'-deoxyadenosine (dAdo).

## Materials and methods

### Chemicals

[2,8-^3^H]Deoxyadenosine 5'-monophosphate ([^3^H]dAMP; 250 μCi; 9.25 MBq; 20 Ci/mmol) was purchased from American Radiolabeled Chemicals, Inc. (St. Louis, MO). [2,8-^3^H]2'-Deoxyadenosine ([^3^H]dAdo; 250 μCi; 9.25 MBq; 32.3 Ci/mmol) was purchased from Moravek Biochemicals (Brea, CA). Nonradioactive 2'-deoxyadenosine and S-(4-nitrobenzyl)-6- thioinosine (NBMPR) were purchased from Wako Pure Chemical Industries (Osaka, Japan). All the other chemicals were purchased from Sigma (St. Louis, MO, USA). The nucleoside compounds dAMP and NBMPR were dissolved in dimethylsulfoxide organic solvent (DMSO). The concentration of DMSO in the final study medium was 0.1% in the presence or absence of inhibitors.

### Cell culture and transfection

Caco-2 cells were maintained in Dulbecco’s modified Eagle’s medium (Sigma, St. Louis, MO, USA) supplemented with 10% fetal bovine serum (ICN Biomedicals Inc., Aurora, OH), 1% non-essential amino acids (Gibco-Invitrogen, Carlsbad, CA), and 100 IU/ml penicillin-100 μg/ml streptomycin (Sigma, St. Louis, MO). The monolayer cultures were grown under an atmosphere of 5% CO_2_-95% air at 37°C. The medium was replaced with fresh medium every 2 days. Upon reaching confluence after 7−10 days in culture, the cells were harvested with 0.25 mM trypsin and 0.2% EDTA (about 5 min at 37°C), resuspended, and seeded on to a new flask.

COS-7 cells were cultured in Dulbecco's modified Eagle's medium supplemented with 10% fetal bovine serum and incubated at 37°C in 5% CO_2_. The open reading frame (ORF) encoding human CNT3 and CD73 were custom-synthesized on order by Eurofins Genomics (Tokyo, Japan). The ORF was subcloned into pcDNA3.1(+) (Invitrogen) by using specific restriction sites HindIII/XhoI (CNT3) and NotI/XhoI (CD73) for expression in COS-7 cells. COS-7 cells were seeded at a density of 2.0 × 10^5^ cells/well on 24-well plastic plates. The cells were transiently transfected on the next day with pcDNA 3.1 construct expressing human CNT3 using Lipofectamine 2000 (Invitrogen) as a transfection reagent, according to the manufacturer’s instructions. COS-7 cells, transfected with pcDNA3.1 empty vector (COS-7/pcDNA3.1), were used as mock cells. CNT3 expression was determined by functional analyses and western blot after 48 h of transfection (S1 Fig). For the transient expression of CNT3 with CD73, COS-7 cells (1.0 × 10^5^ − 2.0 × 10^5^ cells/well initially) were seeded on 24- or 48-well plates, transfected with a plasmid carrying cDNA of CNT3 and the one carrying cDNA of CD73 by using Lipofectamine 2000 reagent, and were cultured for 48 h. The cells were transfected with 0.25−0.5 μg/well of total plasmids in a ratio of 1:1 for CNT3 and CD73. For the expression of CNT3 or CD73 alone, the half of the total amount of plasmids were replaced with the pcDNA3.1 empty vector.

### Uptake study

Caco-2 cells were seeded at a density of 2.5 × 10^5^ cells/well on 24-well plastic plates. The medium of the cell monolayers was refreshed every 2 days, and the monolayers were used for the uptake experiments on day 9−11 after plating. After removal of the culture medium, each well was washed and pre-incubated with an incubation buffer [Hank’s balanced salt saline (HBSS—HEPES buffer (25 mM D-glucose, 137 mM NaCl, 5.37 mM KCl, 0.3 mM Na_2_HPO_4_, 0.44 mM KH_2_PO_4_, 1.26 mM CaCl_2_, 0.8 mM MgSO_4_, 4.17 mM NaHCO_3_, and 10 mM HEPES; pH 7.4 adjusted with 1 M Tris)]. Subsequently, 0.5 ml of the incubation buffer containing a substrate ([^3^H]dAMP) in the absence or presence of various inhibitors were added, and the monolayers were incubated for the indicated time at 37°C. For COS-7 cells, 10 μM NBMPR was included in the incubation buffer to inhibit the endogenous Na^+^ independent nucleoside transporters. Briefly, the cells were washed and pre-incubated with Na^+^-free incubation buffer containing 10 μM NBMPR, and then [^3^H]dAMP, or [^3^H]dAdo was added to initiate the cellular uptake. When required, Na^+^ was isosmotically substituted by N-methyl-d(-)-glucamine. Each cell monolayer was rapidly washed twice with 1.0 ml ice-cold incubation buffer at the end of the incubation period. To quantify the radioactivity of [^3^H]dAMP, or [^3^H]dAdo, the cells were solubilized in 1% SDS/0.2 N NaOH. The remainder of the sample was mixed with 3 ml of scintillation cocktail (Perkin Elmer, Waltham, MA) to estimate the radioactivity. The radioactivity was estimated by a liquid scintillation counter (Packard, 1600TR). All the uptake values were corrected for protein content. The protein concentration was determined using a Pierce® BCA Protein Assay Kit (Thermo Scientific, Waltham, MA, USA) in accordance with the manufacturer's instructions.

### Assay for CD73 by phosphate release

An assay for CD73 activity was performed by estimating the inorganic phosphate (Pi) released from dAMP, as reported previously with minor modifications [21]. The cell monolayer was washed and pre-incubated with phosphate-free buffer (10 mM glucose, 154 mM NaCl, 2 mM KCl, 4 mM MgCl_2_, 18 mM NaHCO_3_, and 25 mM HEPES; pH 7.4 adjusted with 1 M Tris). To estimate CD73 activity in the cell monolayers, dAMP was added to yield a final concentration of 10 μM in each well. The cell monolayers were incubated for the indicated time at 37°C. An aliquot (50 μl) was removed and quickly transferred to a well of a 96-well plate to terminate the reaction. A standard curve of Pi was obtained after every experiment. The volume of the samples in a 96-well plate was made to 100 μl with phosphate-free buffer if necessary, and were treated with 100 μl of BIOMOL GREEN^™^ (Enzo Life Sciences, Farmingdale, NY) reagent and incubated at room temperature for 20 min to allow a green color to develop. The Pi was determined using a multiwell plate spectrophotometer (Hitachi, Tokyo, Japan) at 620 nm. The results of monolayer activity are expressed as pmol of Pi released per 50 μL.

### Data analysis

Nonlinear regression analysis was performed using SigmaPlot 12.5 (HULINKS). The kinetic parameters were calculated using the following equation:

$$V=V_{max}\left[S\right]/\left(K_{m}+\left[S\right]\right)$$

where *V* is the uptake rate of the compounds, *V*_max_ is the maximum uptake rate; [S] is the concentration of the substrate in the medium, and *K*_m_ is the Michaelis-Menten constant.

All experimental data are expressed as the mean ± standard error (S.E.). The mean values were compared by analysis of variance followed by Student’s t-test, Dunnett’s test, and Tukey’s test. A *p* value of < 0.05 was considered as statistically significant.

## Results

### Characterization of dAMP uptake into Caco-2 cells

**Fig 1:**
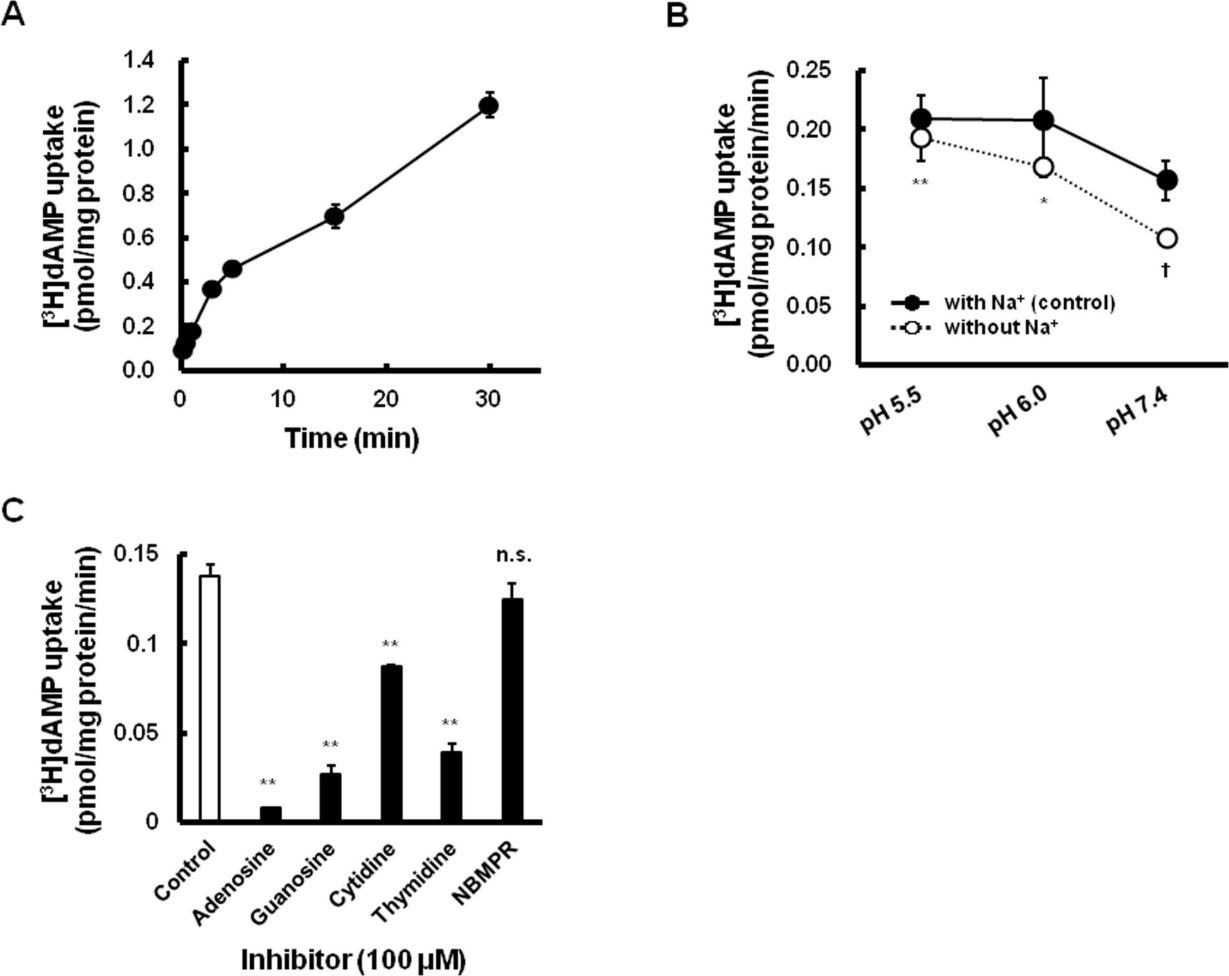
Characterization of [^3^H]dAMP uptake into Caco-2 cells. (A) Time course of [^3^H]dAMP uptake by Caco-2 cells. The uptake of [^3^H]dAMP (2 nM) was estimated at 37°C and pH 7.4. (B) pH- and Na^+^-dependent uptake of [^3^H]dAMP. The uptake of [^3^H]dAMP (2 nM) was estimated at each of the indicated conditions for 3 min. The pH of the transport buffer was varied by appropriately adjusting the concentrations of MES, HEPES, and Tris. When required, Na^+^ was isosmotically substituted by N-methyl-d(−)-glucamine. *, **Significantly different from uptake at pH 7.4 in the absence of Na^+^ at *p* < 0.05, *p* < 0.01, respectively. †Significantly different from uptake at pH 7.4 in the presence of Na^+^ at *p* < 0.05. (C) The inhibitory effects of various compounds on the uptake of [^3^H]dAMP by Caco-2 cells. Caco-2 cells were incubated with [^3^H]dAMP (2 nM) at 37°C and pH 7.4 for 3 min in the absence or presence of each of the indicated compounds (100 μM). **Significantly different from the control at *p* < 0.01. All the data are presented as the mean ± S.E. of at least 3 independent experiments performed in triplicate.

The uptake of [^3^H]dAMP by Caco-2 cells, a cellular model of the human intestinal epithelium, was linear for up to 3 min; and therefore, the uptake was performed with a 3-min incubation period in the subsequent experiments (Fig 1A). The uptake of [^3^H]dAMP decreased with an increase in the extracellular pH from 5.5 to 7.4 under Na^+^-free conditions. Furthermore, the uptake of [^3^H]dAMP under Na^+^-free conditions was significantly decreased in a normal condition at pH 7.4 compared to the uptake of the control (Fig 1B). To elucidate the involvement of the specific types of the nucleoside transporters that are responsible for the uptake of dAMP by Caco-2 cells, we investigated the effects of various compounds, including nucleosides on the initial uptake of [^3^H]dAMP. The uptake was significantly inhibited by purine-nucleosides, typical substrates of CNT2, as well as by pyrimidine-nucleosides, typical substrates of CNT1 (Fig 1C). NBMPR, a specific ENT inhibitor, showed little inhibitory effects on the uptake of [^3^H]dAMP.

### Involvement of CNT3 in the cellular uptake of dAMP

**Fig 2:**
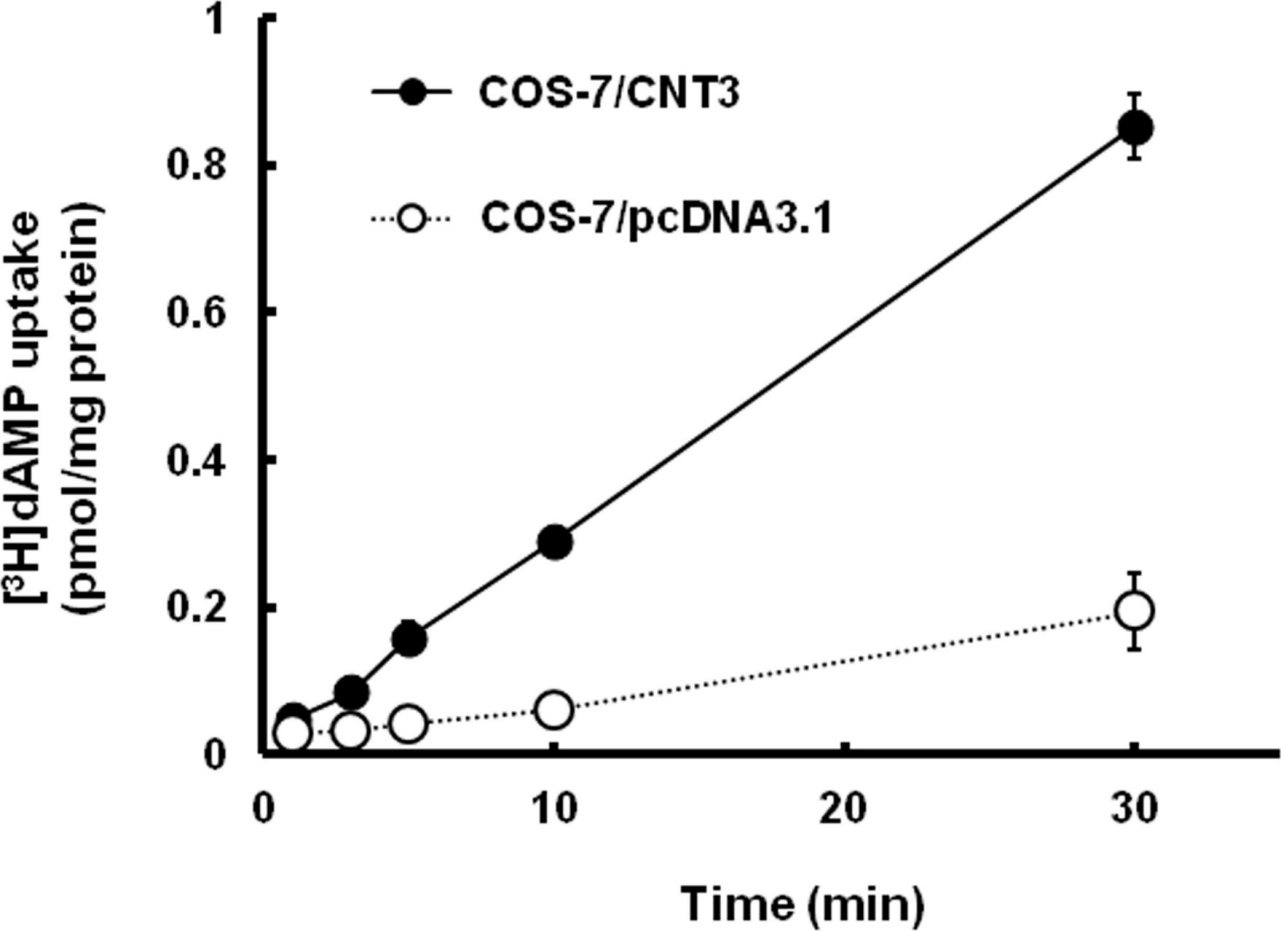
Time course of [^3^H]dAMP uptake into COS-7 cells transiently expressing CNT3. COS-7/CNT3 cells (●) and mock cells (○) were exposed to [^3^H]dAMP (10 nM) at pH 7.4. Each point represents the mean ± S.E. of 3 independent experiments performed in triplicate.

We further examined whether CNT3, which has broad specificity for both pyrimidine and purine nucleoside, was involved in mediating dAMP uptake. Transfection of CNT3 cDNA into COS-7 cells resulted in an increased uptake of [^3^H]thymidine, a typical substrate of CNT3 (S1A Fig). Western blot showed CNT3 protein expression in COS-7 cells transiently expressing CNT3 (COS-7/CNT3) (S1B Fig). The [^3^H]dAMP uptake by COS-7/CNT3 was greater than that by COS-7/pcDNA3.1 and was in a time-dependent manner, indicating that dAMP is a substrate of CNT3 (Fig 2).

### Kinetic analysis of dAMP and dAdo uptake into COS-7/CNT3

**Fig 3:**
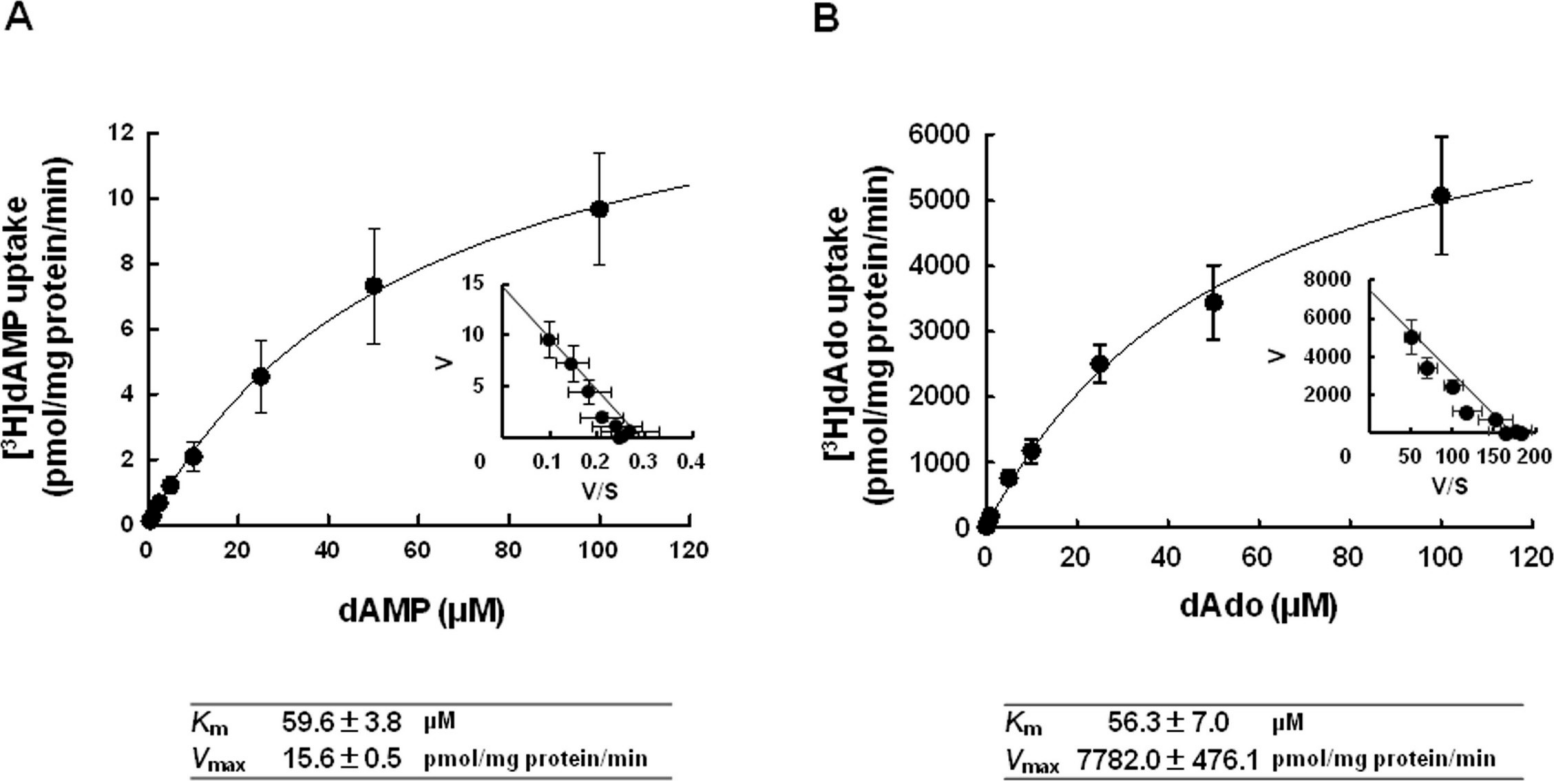
Saturation kinetics of CNT3-mediated uptake of [^3^H]dAMP and [^3^H]dAdo. (A) The uptake of [^3^H]dAMP was estimated over a concentration range of 0.1–100 μM at pH 7.4 for 30 min. (B) The uptake of [^3^H]dAdo was estimated over a concentration range of 0.1–100 μM at pH 7.4 for 30 sec. The specific uptake by CNT3 was estimated by subtracting its uptake by mock cells from that by COS-7 cells transiently expressing CNT3. Inset: Eadie-Hofstee plot. Each point represents the mean ± S.E. of 3 independent experiments performed in triplicate.

As shown in Fig 3A, the concentration dependence of the uptake of dAMP by CNT3 (defined as the difference in uptake between COS-7/CNT3 and COS-7/pcDNA3.1) was determined over a range of concentration (0.5–100 μM). The uptake of dAMP was described by the Michaelis-Menten equation (*K*_m_ = 59.6 ± 3.8 μM, *V*_max_ = 15.6 ± 0.5 pmol·mg protein^–1^min^–1^). The Eadie–Hofstee plot analysis indicated a single saturable component (Fig 3A, inset). Hu et al. have reported that dAdo, which is produced by dephosphorylation of dAMP, is a substrate of CNT3 [22]. The CNT3-mediated uptake of dAdo was saturable, with an apparent *K*_m_ of 56.3 ± 7.0 μM and *V*_max_ of 7782.0 ± 476.1 pmol·mg protein^–1^min^–1^ (Fig 3B). The *K*_m_ value of CNT3-mediated uptake of dAdo was close to that of dAMP whereas the *V*_max_ of dAdo was 500-fold higher than that of dAMP.

### Mutual operation of CNT3 with CD73

**Fig 4:**
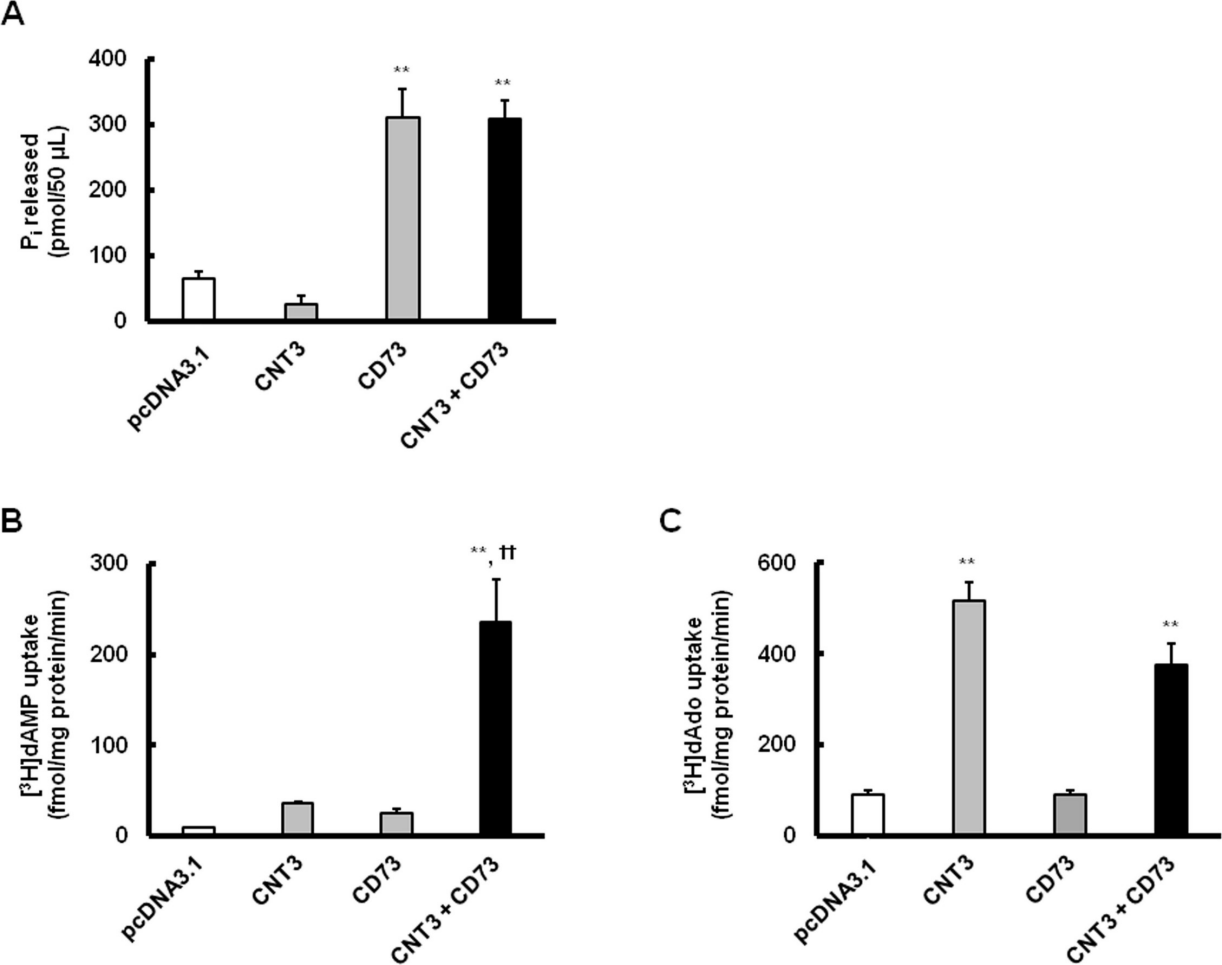
Mutual role of CNT3 with CD73 in [^3^H]dAMP uptake. (A) Activity of CD73 was evaluated at 37°C and pH 7.4 for 30 min in COS-7 cells transfected with a plasmid for CNT3 and another plasmid for CD73 in 1: 1 ratio (0.5 μ g of total plasmid). (B) (C) The uptake of [^3^H]dAMP (10 nM) and [^3^H]dAdo (2 nM) were similarly evaluated in COS-7 cells transfected with a plasmid for CNT3 and another plasmid for CD73. **Significantly different from the control (pcDNA3.1) at *p* < 0.01. ††Significantly different from CNT3 alone at *p* < 0.01. Each column represents the mean ± S.E. of 3 independent experiments performed in triplicate.

To investigate the effects of CD73 on dAMP uptake mediated by CNT3, we conducted a series of uptake experiments using COS-7 cells transfected with CNT3 and CD73. As shown in Fig 4A, the phosphate release from dAMP was significantly increased by the introduction of CD73 into COS-7 cells. [^3^H]dAMP uptake was greater in COS-7 cells expressing CD73 with CNT3 than in those expressing CNT3 alone (Fig 4B). On the other hand, although [^3^H]dAdo uptake was enhanced by the introduction of CNT3 alone, it was not enhanced by the additional introduction of CD73 (Fig 4C). The introduction of CD73 alone in COS-7 cells did not induce any increase in [^3^H]dAMP and [^3^H]dAdo uptake. The introduction of CD73 resulted in a significant increase in [^3^H]dAMP uptake mediated by CNT3, suggesting their mutual role in dAMP uptake.

## Discussion

It has been thought for long that the nucleotides are not directly absorbed from the intestine. Several studies have indicated that purine and pyrimidine ribonucleotides are not absorbed by the everted sacs of rat and hamster intestine [23,24]. On the other hand, Sánchez-Pozo and Gil suggested that the nucleotides are incorporated into the tissues [6]. The molecular mechanisms involved in the absorption of deoxyribonucleotides in the human intestine are, however, unresolved. The present study aimed to elucidate the cellular uptake mechanism of dAMP, particularly the potential involvement of a carrier-mediated mechanism for the intestinal absorption of dAMP.

To characterize the intestinal absorption of dAMP, cellular uptake experiments were conducted using Caco-2 cell monolayers. dAMP was incorporated into Caco-2 cells in a time-dependent manner (Fig 1A). The uptake of dAMP was increased with a drop in pH, suggesting that dAMP transport activity might be linked to the protons (Fig 1B). Furthermore, dAMP uptake was increased in the presence of Na^+^ at a pH of 7.4, while this uptake was completely independent of Na^+^ at a pH of 5.5. These results suggested that dAMP uptake by Caco-2 cells is pH- and Na^+^-dependent. To further characterize the carrier-mediated transport system of dAMP uptake into Caco-2 cells, we examined the inhibitory effects of various compounds, including the substrate of nucleoside transporters, on the uptake of dAMP (Fig 1C). NBMPR (100 μM), a specific ENT inhibitor, had no inhibitory effect on dAMP uptake by Caco-2 cells. The concentration of NBMPR was sufficient to block both ENT1 and ENT2 activity [11]. ENT4 is commonly known as the plasma membrane monoamine transporter (PMAT) which contributes to the apical uptake of metformin in Caco-2 cells [25]. In addition, ENT4/PMAT is also sensitive to NBMPR (*K*_i_ value of 11 μM) [26]. Therefore, these data indicate that the uptake of dAMP in Caco-2 cells does not occur by ENT-mediated transport system. Each nucleoside significantly inhibited the uptake of dAMP. It is well known that CNT1 preferentially transports pyrimidine-nucleoside, whereas CNT2 is a purine-preferring nucleoside transporter. Several studies have shown that CNT1- and CNT2- mediated uridine transport was not inhibited by guanosine and thymidine, respectively [27–29]. Ward et al. have reported that Caco-2 cells lack CNT1 and CNT2 [30]. Another study reported a low level of Na^+^-dependent highly selective nucleoside uptake by Caco-2 cells [31]. Considering that CNT3 transports both pyrimidine and purine nucleosides and is activated not only by Na^+^ but also by H^+^, it is likely that CNT3, rather than CNT1/2, contributes to dAMP uptake into Caco-2 cells [8,9,32].

To investigate the involvement of CNT3 in the transport of dAMP, a transient expression system in the mammalian cells was used in the subsequent experiment. The uptake of dAMP by COS-7/CNT3 cells was significantly higher than that by the mock cells, indicating that dAMP is a substrate of CNT3 (Fig 2). To the best of our knowledge, this study provides the first evidence of direct involvement of nucleoside transporter on deoxyribonucleotide transport. Although CNT3 is expressed in the human gastrointestinal tract, nucleotides are, however, generally considered to be dephosphorylated by phosphatases and nucleotidases in the lumen, and are efficiently absorbed as nucleosides [3,4,33]. It is reported that ecto-5'-nucleotidase/CD73 is the major enzyme responsible for the hydrolysis of nucleotide into nucleoside and phosphate in the human colonic epithelial cell line T84 [21]. The enzyme is also expressed in the apical brush border membranes of Caco-2 cells [34]. The activity of CD73 was estimated by phosphate release from dAMP, and it was found that > 80% of the activity was inhibited by α,β-methylene-ADP (AMP-CP), a known inhibitor of CD73 (S2A and S2B Fig). Western blot showed CD73 protein expression in Caco-2 cells used in our study (S2C Fig). These data suggested that CD73 is the major enzyme which is able to hydrolyze extracellular dAMP on Caco-2 cell monolayers.

As shown in Fig 3, the CNT3-mediated uptake of dAMP and dAdo were saturable and were described by the Michaelis-Menten equation. The *K*_m_ value of CNT3-mediated uptake of dAMP was close to that of dAdo, whereas the *V*_max_ of dAMP was 500-fold less than that of dAdo. The efficiencies of transport of dAMP and dAdo by CNT3 (*V*_max_: *K*_m_ ratio) were 0.26 and 138, respectively. This implied greater CNT3 transportability of dAdo than dAMP and importance of dephosphorylation for the efficient transport of dAdo from dAMP. In this study, we have used dAMP labeled with tritium at the 2- and 8-position of purine nucleus. Considering that dAMP was dephosphorylated by CD73 in the supernatant medium of Caco-2 cells, it is likely to be readily taken up into the cells as dAdo rather than dAMP itself. Therefore, it would appear that dAMP uptake by Caco-2 cells is an example of the phenomenon in which dephosphorylated of luminal nucleotides and carrier-mediated transport of the products are linked to facilitate absorption of the nucleosides into the intestinal mucosa.

Based on the above findings, we hypothesized that the existence of CD73 would facilitate CNT3-mediated intestinal absorption of dAMP. It was assumed that dAMP was converted to dAdo by CD73 before uptake into the cells, thereby facilitating the efficient uptake of dAdo. In support of this hypothesis, we showed that dAMP is directly dephosphorylated by CD73, and CNT3-mediated uptake of dAMP (^3^H-labeled at the 2- and 8-position of purine nucleus) could be facilitated by the introduction of CD73. Several studies suggest that COS-7 cell line exhibits a very low basal level of nucleotidase activity [35,36]. As shown in Fig 4A, the phosphate release from dAMP was significantly increased by the introduction of CD73 into COS-7 cells, whereas it was slightly decreased by the introduction of CNT3 alone. These results might be explained by the CD73-mediated dephosphorylation of dAMP and CNT3-mediated cellular uptake. Furthermore, dAMP uptake was significantly increased by the introduction of CD73 with CNT3 than in cells expressing CNT3 alone (Fig 4B). On the other hand, although dAdo uptake was enhanced by the introduction of CNT3 alone, it was not enhanced by the additional introduction of CD73 (Fig 4C). These data suggest that CNT3 mediates the intestinal absorption of dAdo from dAMP along with CD73. Interestingly, [^3^H]AMP uptake was also greater in COS-7 cells expressing CD73 with CNT3 than in those expressing CNT3 alone (S3 Fig). Therefore, the facilitation of cellular uptake via CNT3 by the additional introduction of CD73 may occur not only in dAMP but also in AMP, a ribonucleotide. In addition, enhanced cellular uptake of [^3^H]AMP with the introduction of CNT3 alone was greater than that of [^3^H]dAMP, suggesting that AMP is a better substrate of CNT3 than dAMP. As shown in Fig 3, the affinity of dAdo for CNT3 (*K*_m_: 56 μM) was lower than that of adenosine obtained from previously published data (*K*_m_: 15 μM) [34]. Thus, a hydroxyl group (-OH) at the 2' position of the ribose sugar moiety is assumed to play an important role in the recognition of substrates by CNT3. The other CNT members (CNT1 and CNT2) have been reported to be located in the apical side of the enterocytes, and are involved in the transport of dAdo [27,37–39]. Therefore, these transporters may also be involved in the intestinal absorption of dAMP along with CD73.

The luminal pH in the proximal small intestine ranges from 5.5 to 7.0 and gradually increases and reach up to 7.5 in the terminal ileum [40]. As shown in S4 Fig, western blot analysis using normal human samples showed that CNT3 is expressed all along the small intestine. CD73 was also detected at the protein level in the duodenum and ileum, and at a much reduced level in the jejunum. Although the reason for the weak detection in the jejunum is unclear, it was revealed that CNT3 and CD73 are co-expressed at the protein level, at least in the duodenum and ileum. Given that CD73 has a neutral pH optimum [41], efficient absorption of dAdo following dephosphorylation of dAMP by CD73 is likely to be increased in the terminal ileum. Further studies are needed to elucidate the mutual role of CNTs and CD73 in utilization and intestinal absorption of deoxyribonucleotides *in vivo*.

In conclusion, our current study demonstrates that the cellular uptake of dAMP in Caco-2 cells is mediated at least partly by CNT3, a broad-specificity nucleoside transporter. We have also shown that ecto-5'-nucleotidase/CD73 can catalyze the dephosphorylation of extracellular dAMP. Additionally, we found that the efficiency of dAMP transport by CNT3 is much less than that of dAdo. Therefore, CNT3 contributes to the uptake of dAMP in the absorption process of intestinal epithelial cells by functioning in cooperation with CD73, thereby facilitating efficient absorption of dAdo from the intestinal lumen.

## Supporting information

S1 Fig Characterization of COS-7 cells transiently expressing CNT3. (A) Uptake of [^3^H]thymidine (10 nM) by COS-7 cells transiently expressing CNT3. COS-7/CNT3 cells and mock cells were exposed to 10 nM [^3^H]thymidine at pH 7.4 for 30 min in the presence of 10 μM NBMPR. Each column represents the mean with S.D. of 3 measurements. (B) Expression of CNT3 was assessed by western blot. Whole cell extracts were prepared and resolved using SDS-PAGE. Western blot was carried out with antibodies against CNT3 (Abcam, ab223085, 1:2000), and β-actin (Millipore, MAB1501, 1/1000).
(TIF)

S2 Fig Activity of CD73 in Caco-2 cells. (A) Time course of dAMP hydrolysis in Caco-2 cells. The cells were incubated with 10 μM dAMP for the indicated time at 37°C and pH 7.4. (B) Effects of AMP-CP, an ecto-5'-nucleotidase (CD73) inhibitor, on dAMP hydrolysis. The cells were incubated with 10 μM dAMP at 37°C and pH 7.4 for 5 min in the absence or presence of AMP-CP. *, **Significantly different from control (0 μM) at *p* < 0.05, *p* < 0.01, respectively. All data are presented as the mean ± S.E. of at least three independent experiments performed in triplicate. (C) Expression of CD73 was assessed by western blot. Whole cell extracts were prepared and resolved using SDS-PAGE. Western blot was performed with antibodies against CD73 (Santa Cruz, sc32299, 1:1000), and β-actin (Millipore, MAB1501, 1/1000).
(TIF)

S3 Fig Mutual role of CNT3 with CD73 in [3H]AMP uptake. [2,8-^3^H]Adenosine 5'-monophosphate ([^3^H]AMP; 250 μCi; 9.25 MBq; 20 Ci/mmol) was purchased from American Radiolabeled Chemicals, Inc. The uptake of [^3^H]AMP was evaluated in COS-7 cells transfected with a plasmid for CNT3 and another plasmid for CD73. The cells were incubated with [^3^H]AMP (10 nM) at 37°C and pH 7.4 for 30 min in the presence of 10 μM NBMPR. *, **Significantly different from the control (pcDNA3.1) at *p* < 0.05, *p* < 0.01, respectively. ††Significantly different from CNT3 alone at *p* < 0.01. Each column represents the mean ± S.E. of four independent experiments performed in triplicate.
(TIF)

S4 Fig Expression of CNT3 and CD73 protein along the normal human small intestine. Western blot analysis was performed using total proteins isolated from the duodenum (BioChain, P1234101), jejunum (BioChain, P1234230) and ileum (BioChain, P1234227) of organ donors. Western blot was performed with antibodies against CNT3 (Abcam, ab223085, 1:2000), CD73 (Santa Cruz, sc-32299, 1:1000), and β-actin (Millipore, MAB1501, 1/1000).
(TIF)
