# Supplementary figures and images for: Mutual role of ecto-5'-nucleotidase/CD73 and concentrative nucleoside transporter 3 in the intestinal uptake of dAMP
Source: PLoS One. 2019 Oct 21;14(10):e0223892. doi: 10.1371/journal.pone.0223892 (PMC6802847; doi:10.1371/journal.pone.0223892)

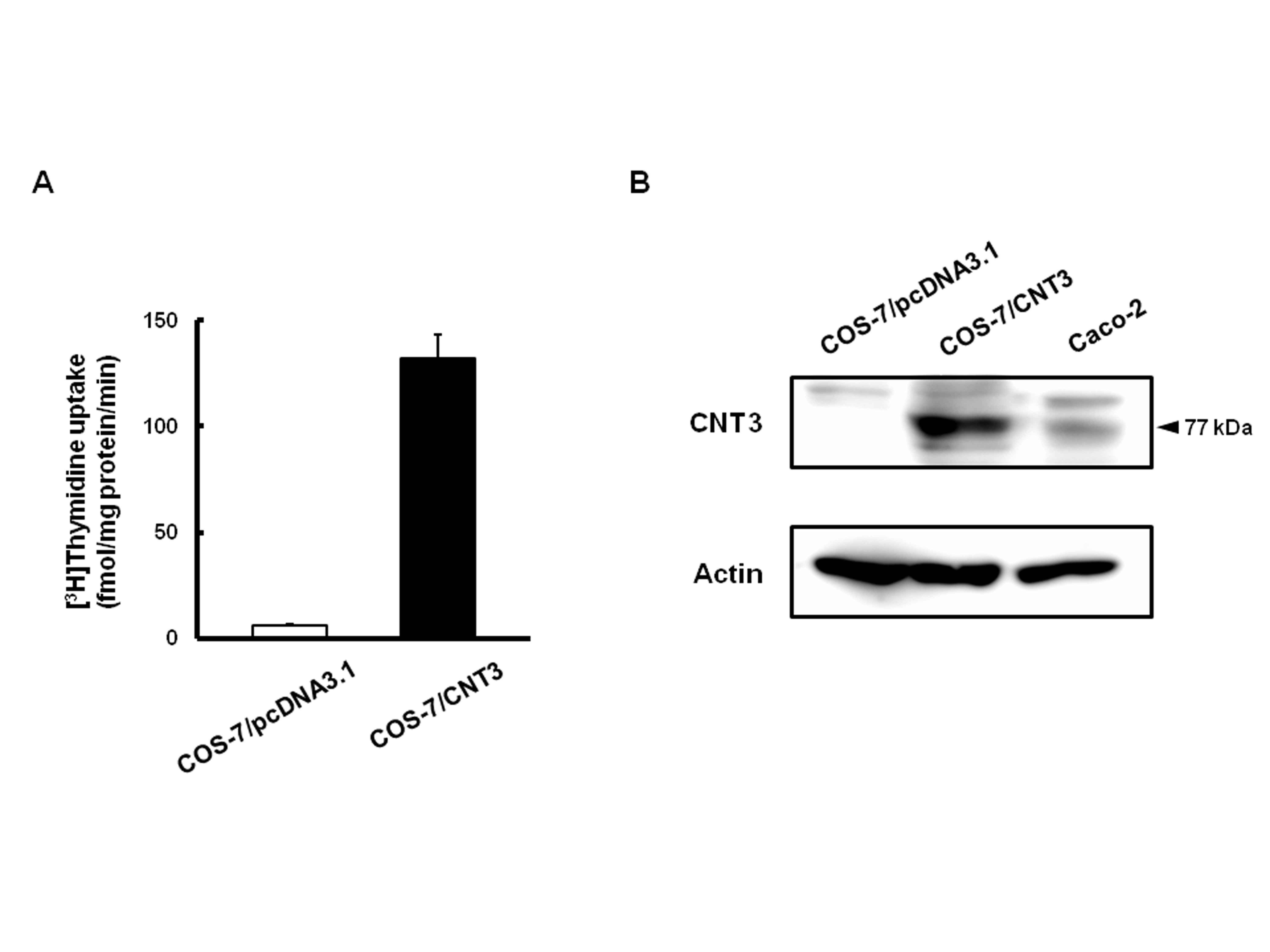

Supplement: S1 Fig — (A) Uptake of [3H]thymidine (10 nM) by COS-7 cells transiently expressing CNT3. COS-7/CNT3 cells and mock cells were exposed to 10 nM [3H]thymidine at pH 7.4 for 30 min in the presence of 10 μM NBMPR. Each column represents the mean with S.D. of 3 measurements. (B) Expression of CNT3 was assessed by western blot. Whole cell extracts were prepared and resolved using SDS-PAGE. Western blot was carried out with antibodies against CNT3 (Abcam, ab223085, 1:2000), and β-actin (Millipore, MAB1501, 1/1000). (TIF) [file pone.0223892.s001.TIF]

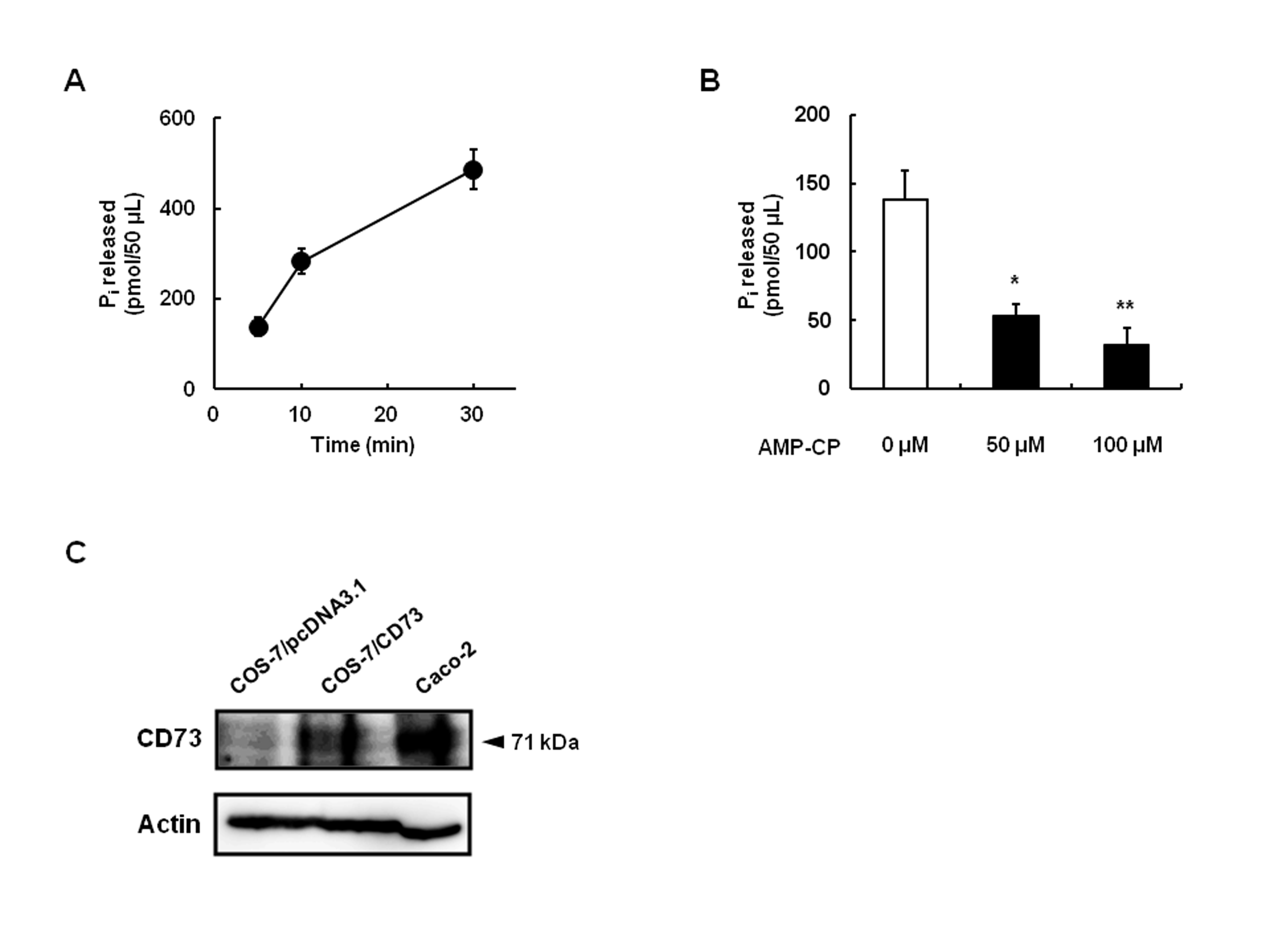

Supplement: S2 Fig — (A) Time course of dAMP hydrolysis in Caco-2 cells. The cells were incubated with 10 μM dAMP for the indicated time at 37°C and pH 7.4. (B) Effects of AMP-CP, an ecto-5'-nucleotidase (CD73) inhibitor, on dAMP hydrolysis. The cells were incubated with 10 μM dAMP at 37°C and pH 7.4 for 5 min in the absence or presence of AMP-CP. *, **Significantly different from control (0 μM) at p < 0.05, p < 0.01, respectively. All data are presented as the mean ± S.E. of at least three independent experiments performed in triplicate. (C) Expression of CD73 was assessed by western blot. Whole cell extracts were prepared and resolved using SDS-PAGE. Western blot was performed with antibodies against CD73 (Santa Cruz, sc32299, 1:1000), and β-actin (Millipore, MAB1501, 1/1000). (TIF) [file pone.0223892.s002.TIF]

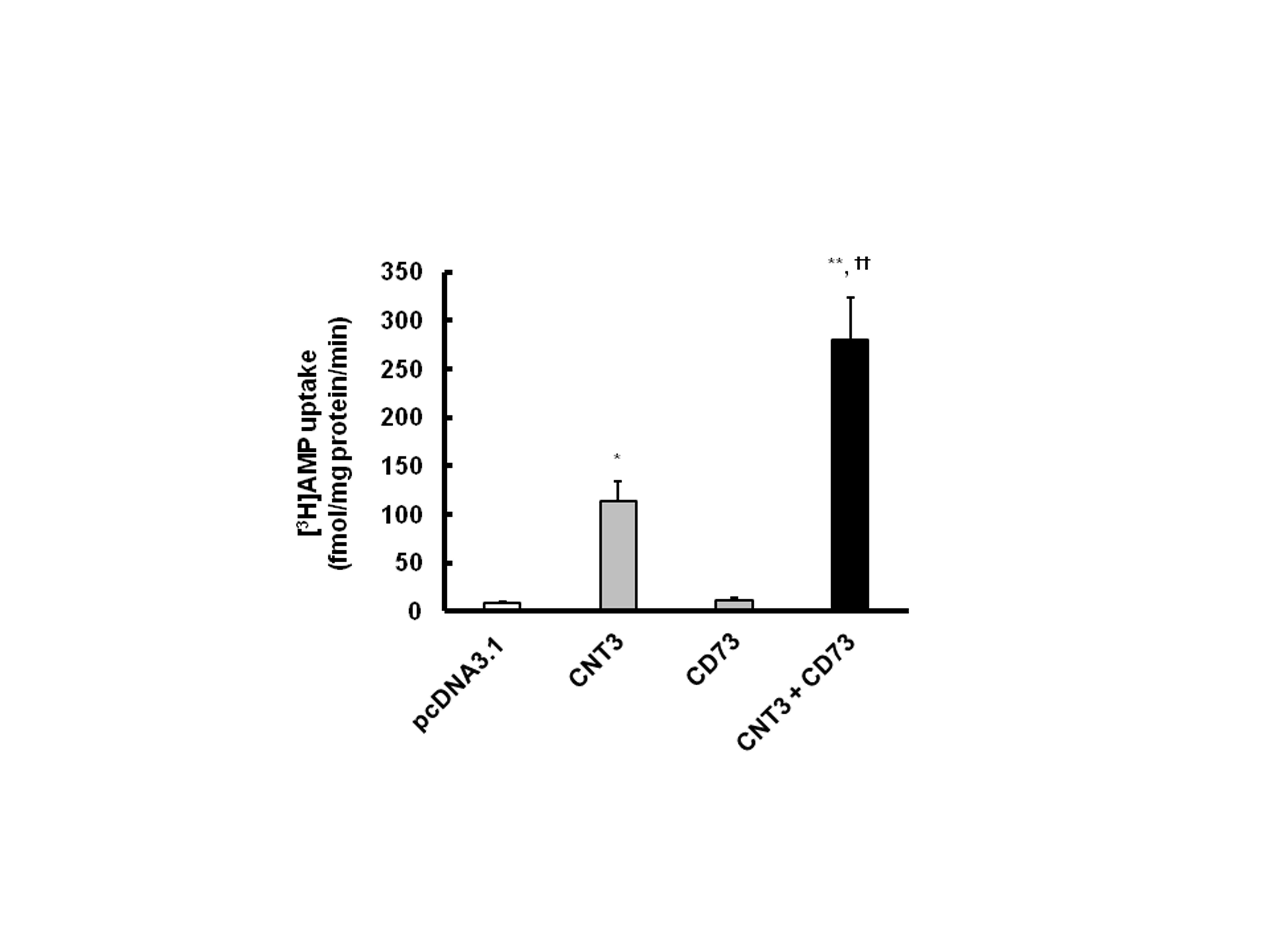

Supplement: S3 Fig — [2,8-3H]Adenosine 5'-monophosphate ([3H]AMP; 250 μCi; 9.25 MBq; 20 Ci/mmol) was purchased from American Radiolabeled Chemicals, Inc. The uptake of [3H]AMP was evaluated in COS-7 cells transfected with a plasmid for CNT3 and another plasmid for CD73. The cells were incubated with [3H]AMP (10 nM) at 37°C and pH 7.4 for 30 min in the presence of 10 μM NBMPR. *, **Significantly different from the control (pcDNA3.1) at p < 0.05, p < 0.01, respectively. ††Significantly different from CNT3 alone at p < 0.01. Each column represents the mean ± S.E. of four independent experiments performed in triplicate. (TIF) [file pone.0223892.s003.TIF]

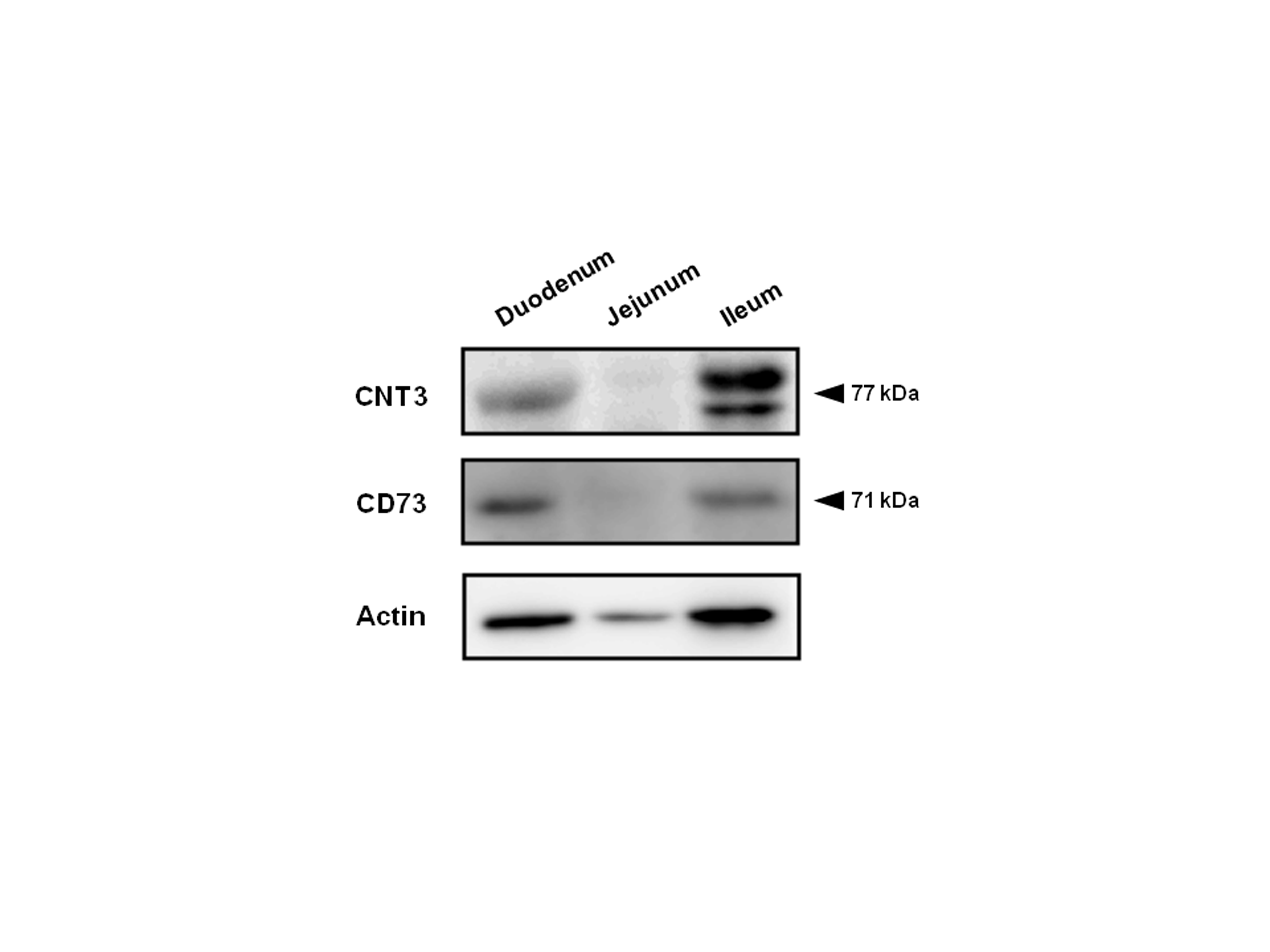

Supplement: S4 Fig — Western blot analysis was performed using total proteins isolated from the duodenum (BioChain, P1234101), jejunum (BioChain, P1234230) and ileum (BioChain, P1234227) of organ donors. Western blot was performed with antibodies against CNT3 (Abcam, ab223085, 1:2000), CD73 (Santa Cruz, sc-32299, 1:1000), and β-actin (Millipore, MAB1501, 1/1000). (TIF) [file pone.0223892.s004.TIF]
